# Permutation Entropy of Weakly Noise-Affected Signals

**DOI:** 10.3390/e24010054

**Published:** 2021-12-28

**Authors:** Leonardo Ricci, Antonio Politi

**Affiliations:** 1 Department of Physics, University of Trento, 38123 Trento, Italy; 2 Center for Mind/Brain Sciences (CIMeC), University of Trento, 38068 Rovereto, Italy; 3 Institute for Pure and Applied Mathematics and Department of Physics, University of Aberdeen, Aberdeen AB24 3UE, UK

**Keywords:** entropy, ordinal patterns, multifractal analysis

## Abstract

We analyze the permutation entropy of deterministic chaotic signals affected by a weak observational noise. We investigate the scaling dependence of the entropy increase on both the noise amplitude and the window length used to encode the time series. In order to shed light on the scenario, we perform a multifractal analysis, which allows highlighting the emergence of many poorly populated symbolic sequences generated by the stochastic fluctuations. We finally make use of this information to reconstruct the noiseless permutation entropy. While this approach works quite well for Hénon and tent maps, it is much less effective in the case of hyperchaos. We argue about the underlying motivations.

## 1. Introduction

The analysis of ordinal patterns is one of the most used tools to characterize irregular (whether stochastic or deterministic) signals [1]. Instead of decomposing a given signal in terms of templates defined a priori, as, for example, in the case of Fourier modes, one lets the time series suggest the relevant motifs of a given length *m*.

Possibly the most relevant known quantifier that can be built by classifying ordinal patterns is the so-called permutation entropy (PE) [2], which quantifies the diversity of motifs, or symbolic sequences, by encoding *m*-dimensional sections of a time series into permutations. The growth rate of PE with *m* coincides asymptotically (i.e., for m→∞) with the Kolmogorov–Sinai (KS) entropy, thus giving appeal to this concept, which does not require the definition of any preassigned partition of the phase space. The possibility of straightforwardly computing PE out of the observed occurrence frequency of symbolic sequences is balanced by it being heavily affected by finite-size corrections. They are due to the entropy increase originating from the implicit refinement of the phase-space partition when the window length is increased (a phenomenon entirely different from the entropy increase associated with a finite KS entropy). These spurious effects can be either removed by assuming an a priori knowledge of the fractal structure of the underlying attractor [3], or by reconstructing, for any given value of *m*, the Markov process that describes the time succession of symbolic sequences [4].

The increasing rate of PE is a well-defined concept whenever the time series is strictly deterministic. However, experimental signals are typically affected by noise, which may be so strong as to mask the underlying chaotic nature. For this reason, attention has been devoted to methods to distinguish more or less stochastic processes. In a notable attempt [5], it was proposed to accompany the estimate of PE with a second quantifier that measures the distance between the observed distribution of the occurrence probabilities of the symbolic sequences from the uniform distribution expected for a perfectly random process.

In this paper, we focus on deterministic signals affected by a weak observational, i.e., additive, noise, with the goal of unveiling general scenarios and possibly using them as a springboard to tackle more complex problems. For this purpose, we study here four dynamical systems: (i) the Hénon map, as one of the prototypical examples of chaotic dynamics; (ii) the tent map, a simple model that offers chances to derive analytical results; (iii) the Hénon map with delay, also referred to as the generalized 3D Hénon map, which makes up a hyperchaotic system to investigate the generality of the scenario; and (iv) the Mackey–Glass model, which provides a more realistic continuous time system.

We first quantify the effect of additional noise by determining the PE for different values of the window length *m* and the noise amplitude σ. We find that, in the small noise limit, the entropy increases according to a power law as ${\left(\sigma m^{\alpha }\right)}^{k}$, where the exponents α, *k* depend on, and are therefore characteristic of, the underlying dynamical system. A multifractal analysis is then implemented, where the number of symbolic sequences characterized by (approximately) the same probability is determined. As a result, the whole population can be split into two “phases”, the former consisting of the original, truly deterministic, symbolic sequences and the latter of new symbolic sequences, which are noise-induced by “twisting” the original ones. Finally, a more quantitative analysis is performed to clean the noise-induced sequences via a supervised approach that allows assembling them back to their respective “ancestor” sequences. This cleaning process yields a decrease in the PE, which comes closer to the expected, purely deterministic value.

The paper is organized as follows. The scaling analysis of the PE increase as a function of the window length and noise amplitude is discussed in Section 2, while the multifractal analysis is introduced and illustrated in Section 3. The attempted reconstruction of the deterministic value of entropy upon reassignment of noise-induced symbolic sequences is the topic of Section 4, while the remaining open problems are presented in the final Section 5.

## 2. Permutation Entropy and Its Noise-Induced Scaling Behavior

Let $\left\{x_{n}\right\}$ denote a sequence of scalar variables, which may either arise from the iteration of a discrete-time map, or by sampling a continuous-time signal. In this paper, four dynamical systems are considered:

the Hénon map,

$$x\left(n+1\right)=a-x^{2}\left(n\right)-bx\left(n-1\right)\;,\;\mathrm{with}\;a=1.4,\;b=0.3,\;n\in Z\;;$$

the tent map,

$$x\left(n+1\right)=2\;\min\left\{x(n),\;1-x(n)\right\}\;,\;\mathrm{with}\;n\in Z,\;x\left(n\right)\in \left[0,\;1\right]\;;$$

the generalized 3D Hénon map,

$$x\left(n+1\right)=a-x^{2}\left(n-1\right)-bx\left(n-2\right)\;,\;\mathrm{with}\;a=1.5,\;b=0.29,\;n\in Z\;;$$

and the Mackey–Glass model,

$$\frac{x(t)}{dt}=a\frac{x(t-t_{d})}{1+x^{c}\left(t-t_{d}\right)}-bx\left(t\right)\;,\;\mathrm{with}\;a=2,\;b=1,\;c=10,\;t_{d}=3.3\;.$$

In the case of the tent map, to avoid the well-known issue of periodicity due to the finite precision of floating-point representation, a realization of a random Gaussian variate $∼N\left(0,\;\sigma_{\mathrm{tm}}^{2}\right)$, with $\sigma_{\mathrm{tm}}=10^{-12}$, is dynamically added to the outcome x(n) at step *n*. If the result of this last operation is >1 (<0), an amount equal to 1 is further subtracted (added) in order to keep the final result within the interval [0,1]. However, due to the standard deviation of the Gaussian perturbation and the length of the computed time series (see below), these corrections are definitely unlikely (p≪1%). In the case of the Mackey–Glass model, the integration of the differential equation is carried out via a Runge–Kutta fourth-order algorithm upon sampling the *x* variable with a unitary sampling rate (T=1). In all cases, the starting point is randomly set, and the first $10^{3}$ evolution steps are discarded from further analysis to avoid transient effects. The length of each sequence is $4\times 10^{9}$. Due to this large value, random fluctuations of the different parameters numerically assessed out of the time series are deemed to be negligible [6,7]. The average value of the *x* variables are 0.36, 0.5, 0.39, and 0.9, respectively.

Let $X_{n}\equiv \left(x_{n},\;x_{n+1},\ldots ,\;x_{n+m-1}\right)$ be a trajectory—or window—composed of *m* consecutive observations. According to the procedure devised by Bandt and Pompe [2], the window $X_{n}$ is encoded as the symbolic sequence (permutation), henceforth also referred to as word, $W_{n}=\left(w_{n,1},\;w_{n,2},\ldots ,\;w_{n,m}\right)$, where the integer $w_{n,j}$ belongs, like the index *j*, to the range [1,m] and corresponds to the rank, from the smallest to the largest, of $x_{n+j-1}$ within $X_{n}$. In the unlikely event of an equality between two or more observations, the relative rank is provided by the index *j*. This way, the very same word *W* encodes trajectories all exhibiting the same temporal pattern, without paying attention to the actual values taken by the variables $x_{n}$. Assuming a sufficiently long time series and denoting with $p_{W}$ the rate with which a word *W* is observed therein, PE is defined as

(1) $$H\left(m\right)=-\sum_{W}p_{W}\log p_{W}\;,$$

where the sum is extended on the set of all possible words, whose cardinality is m! (we assume here 0log0=0).

**Figure 1:**
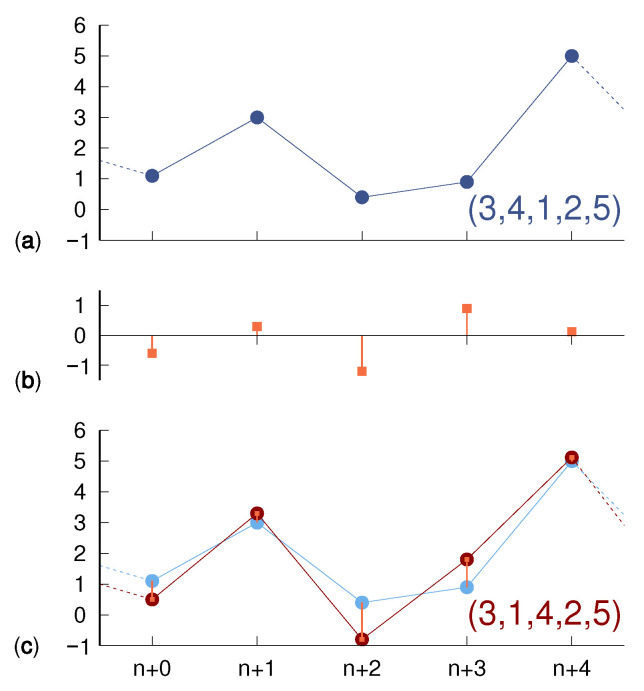
(**a**) According to the rule described in the main text, a sample noiseless trajectory (1.1,3,0.4,0.9,5) of dimension m=5 is encoded as (3,4,1,2,5). The additive contribution of noise, e.g., (−0.6,0.3,−1.2,0.9,0.12) as shown in (**b**), yields the noise-affected trajectory (**c**) given by (0.5,3.3,−0.8,1.8,5.12), whose encoding is (3,1,4,2,5).

In order to investigate the variation of PE due to the presence of a weak observational noise, we add to each observation $x_{n}$ a realization of a random variable $\xi_{n}$ uniformly distributed within the interval $\left[-\sqrt{3}\sigma ,\;\sqrt{3}\sigma \right]$ so that its standard deviation is equal to σ. Observational noise is effective insofar that it modifies the ordering of the $x_{n}$ values in a given window. In the example of Figure 1, a noiseless trajectory, encoded as (3,4,1,2,5), yields, under the effect of random fluctuations, a new trajectory, which is now encoded as (3,1,4,2,5): the neighboring symbols 1 and 4 are swapped due to their initial separation 0.2 being overcome by “counterpropagating” shifts induced by observational noise. This leads to a diffusion of the observed occurrence frequency of a given sequence toward neighboring ones, including those that might go unvisited in the absence of noise, and therefore induces an entropy increase ΔH(m,σ) given by

ΔH(m,σ)=H(m,σ)−H(m,0).

In this equation, H(m,σ) is the PE computed according to Equation (1) on rates $p_{W}\left(\sigma \right)$ observed on a time series affected by a noise of amplitude σ, whereas H(m,0) corresponds to the noiseless case.

One expects the entropy increase to depend on the noise strength σ rescaled to the typical separation Δx between consecutive values along the window (after reordering) since Δx represents the size of the “barrier” to be overcome in order for the encoding of the ordinal pattern to change. By considering that the variables of our dynamical systems are all of order O(1), we expect Δx≈1/m (more precisely 1/(m−1)), and we therefore expect the effective noise to be equal to σm.

**Figure 2:**
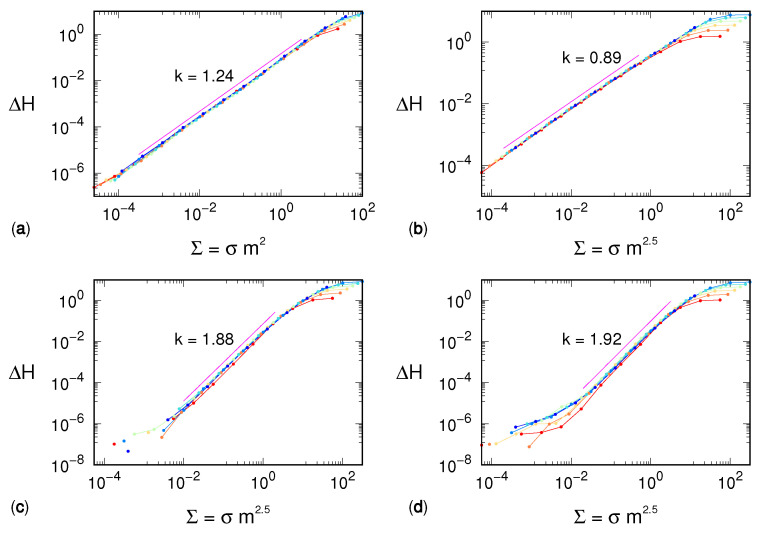
Permutation entropy variation ΔH as a function of the effective noise $\Sigma =\sigma m^{\alpha }$ in the case of (**a**) Hénon map, (**b**) generalized 3D Hénon map, (**c**) tent map, and (**d**) Mackey–Glass model. The exponent α is 2 for the Hénon map and 2.5 for the three other dynamical systems. In each case, the line colors, ordered according to a rainbow palette, refer to different values of *m*, from 5 (red) to 11 (blue). The magenta line corresponds to the result of a fit of Equation (2) to all curves at different values of *m*. The line is upward displaced for graphical reasons, and its projection on the horizontal axis corresponds to the range used for the fit procedure. While the power law *k* exponents are reported on the respective graphs, the factors *b* are equal to −1.1, −0.4, −1.5, and −1.5, respectively.

Simulations performed for different noise amplitudes and window lengths reveal a different scenario. The entropy increase depends on $\Sigma \equiv \sigma m^{\alpha }$, as clearly attested by the very good “data collapse” shown in Figure 2, which extends over a wide range of scales for each one of the four selected models.

The exponent α is not only model-dependent—being equal to 2 for the Hénon map, and approximately to 2.5 for the three other dynamical systems—but it is strictly larger than 1. This observation suggests that our mean-field argument is too naive and the distribution of separations between consecutive variables must be included in the reasoning. We are then driven toward considerations similar to those that allowed refining the estimate of the KS entropy in Ref. [3], though it is not clear how to transform them into quantitative arguments.

An additional remarkable result revealed by our simulations is that in all cases, the entropy increase scales as

(2) $$\Delta H\left(m,\sigma \right)≅b\;\Sigma^{k}\;,$$

where both the factor *b* and the exponent *k* are distinctive features of the underlying dynamical system. The lower quality of the collapse for small values of Σ is most likely an effect of poor statistics: the smaller the deviation, the larger the number of points required to compute significant differences between the populations of the symbolic sequences. On the other side, namely for large values of Σ, the overlap between curves at different *m* values has to stop due to the finiteness of H(m), which cannot exceed logm!∼mlog(m/e) and thus leads to *m*-dependent saturation.

We also note that the exponent *k* increases with the dynamical complexity of the signal. We can argue as follows: noise increases the entropy since it redistributes the mass of each symbolic sequence toward the neighboring ones. In the following sections, we investigate this redistribution.

## 3. Multifractal Analysis

In the previous section, we described how noise affects entropy. Here, we focus on a more detailed description by reconstructing the distribution of the different words for different noise amplitudes. This is essentially an application of the so-called multifractal formalism [8]. We consider a single *m* value, namely, m=10: this choice, while being still computationally manageable, provides a sufficiently large number of possible symbolic sequences (≈3.6 $\times 10^{6}$) so that the related set can be considered as being continuous.

For a given noise amplitude σ, we first build a histogram where the occupation $N_{i}$ of the *i*-th bin is given by the number of different symbolic sequences whose logarithm of the observed probability lies in the interval $\left[\log p_{i},\;\log p_{i}+\delta \right]$ (δ is the logarithmic scale bin width):

(3) $$N_{i}=\#\left\{W|\log p_{i}⩽\log p_{W}<\log p_{i}+\delta \right\}\;.$$

Unvisited symbolic sequences are not taken into account. Because all simulations were carried out by analyzing time series consisting of $4\times 10^{9}$ points, the minimum possible value of $\log p_{W}$ is approximately −22.1. The histograms were thus constructed by splitting the range [−23,−3) into 25 bins, so that δ=0.8. (This choice essentially corresponds to the result of the application of Freedman–Diaconis’ rule).

**Figure 3:**
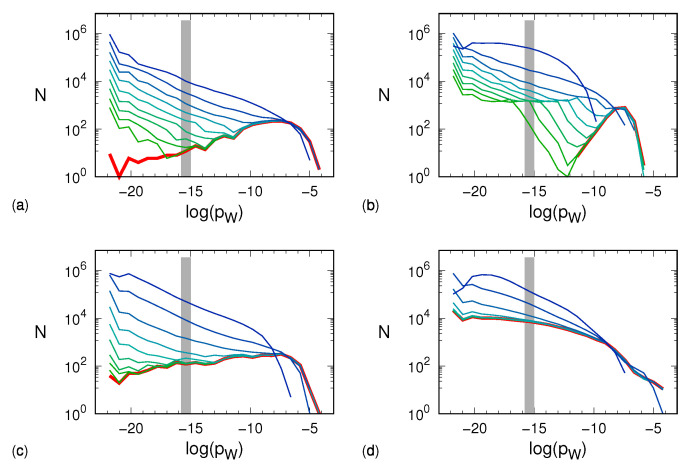
Histograms, computed according to Equation (3), of the observed, nonvanishing probabilities of the different words for different noise amplitudes and obtained by setting m=10: (**a**) Hénon map, (**b**) tent map, (**c**) generalized 3D Hénon map, (**d**) Mackey–Glass model. For each dynamical system, the red line corresponds to the noiseless histogram, whereas the other lines correspond to histograms computed in the presence of different noise amplitudes σ. The line color palette veers from green, corresponding to $\sigma =10^{-5}$, to blue, corresponding to $\sigma =10^{-1}$, with multiplicative steps of σ=1012≈3.2. The bin width is 0.8. Each gray area shows the histogram when the symbolic sequences are equiprobable so that the bin including $\log p_{W}=-\log m!\approx -15.1$ becomes populated with $10!\approx 3.6\times 10^{6}$ symbolic sequences. This situation occurs when the dynamics is purely stochastic and thus corresponds to the distribution with σ→∞.

The results are plotted in Figure 3 for the four different models and different noise amplitudes. It is worth remarking that the logarithmic binning used in the analysis of this section is both an ingredient of the multifractal formalism and a way to cope with the interval of occurrence probabilities, which covers about eight decades. We also note that the sum of the bin populations—and thus a fortiori the population of each single bin—cannot exceed $m!=10!\approx 3.6\times 10^{6}$. This observation is relevant when considering the progressive effect of noise on the distributions of the different words.

The data in panel (a) refer to the Hénon attractor. The red curve corresponds to the zero-noise limit in which we see a relatively small number of unpopulated words. As long as the noise is small (≲$10^{-2}$), its effect mostly turns out to yield a large number of very lightly populated sequences, whereas the distribution of heavier sequences is basically untouched. Quantitative changes can be appreciated when the noise amplitude reaches 0.1. The tent map exhibits a similar scenario. Here, the zero-noise histogram is well confined between $\log p_{W}∼-11$ and $\log p_{W}∼-6$. At a noise amplitude of ∼$10^{-2}$, the noise-induced probability shuffling becomes so effective as to erode and leftward shift the most populated bins of the noiseless distribution. The same erosion is visible in the plots of the generalized 3D Hénon map, which, on the lower side, shows a behavior more similar to that of the Hénon map. The Mackey–Glass model reveals a partially different scenario. In the noiseless case, light boxes are more “popular” than for the other dynamical systems, which makes the distribution more robust to weak noise. However, at a noise amplitude of ∼$3\times 10^{-2}$, both the erosion and the leftward shift of the most populated bins set on. Altogether, we see that the more or less pronounced presence of a low-probability tail in the various distributions reflects in a smaller or larger robustness to noise (the *k* exponent introduced in the previous section).

**Figure 4:**
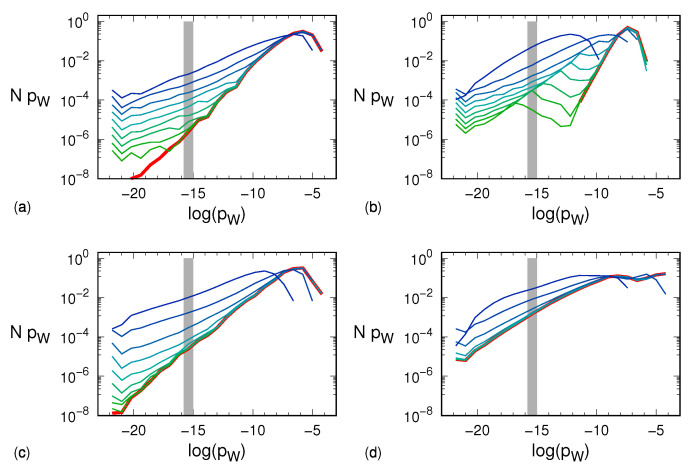
Bin population $N_{i}$ of the respective histograms of Figure 3 multiplied times $\exp(\langle \log p_{i}\rangle )$, namely the probability $p_{W}$ corresponding to the center of the *i*-th logarithmic scale bin: (**a**) Hénon map, (**b**) tent map, (**c**) generalized 3D Hénon map, (**d**) Mackey–Glass model. Line and color notation is the same as explained in the caption to Figure 3. Similarly, each gray area shows the histogram when the symbolic sequences are equiprobable: the bin including $\log p_{W}=-\log m!\approx -15.1$ thus becomes populated with 10! symbolic sequences, each having a probability ${\left(10!\right)}^{-1}$ so that Np=1. This situation occurs when the dynamics is purely stochastic and thus corresponds to the distribution with σ→∞. For the noise amplitudes taken into account, the tent map is apparently the fastest to approach that limit behavior.

The same data shown in Figure 3 can be presented by multiplying *N* by *p* so as to have a measure of the actual weight of sequences with probability *p*. The results are plotted in Figure 4, where one can indeed appreciate the smallness of the contribution arising from the very large number of light sequences.

## 4. Assessing the Entropy Increase via Distribution Reconstruction

As already argued in the previous section, observational noise increases the entropy both because it induces new sequences and because it “democratizes” their distribution. In this section, we discuss a method to possibly minimize the entropy increase. The method is based on the idea of moving back the content of the new sequences to the corresponding “ancestors”. The method is supervised, as knowledge of the symbolic sequence occurring in the zero-noise case is assumed. The ancestor of a new sequence is a noiseless sequence that is identified according to a suitable distance criterion, as follows.

Several definitions of distance between permutations have been proposed [9]. One possibility is the Hamming distance, which corresponds to number of positions in which the two permutations differ. Another possibility is the transposition distance [10], namely the minimum number of swappings between non-necessarily contiguous positions to make the first permutation coincide with the second. We use here the so-called deviation distance [11], which is a metric and is computed in a number of steps O(m). Given the two words $U=(u_{1},u_{2},\ldots ,u_{m})$ and $V=(v_{1},v_{2},\ldots ,v_{m})$, their deviation distance is

$$d\left(U,V\right)=\frac{1}{2}\sum_{i,j}\delta_{u_{i},v_{j}}\left|i-j\right|\;,$$

where $\delta_{u_{i},v_{j}}$ is the Kronecker delta. In practice, for each symbol in the first word, we search for the position of the same symbol in the second word, we determine the absolute separation between the two positions, and we sum all of them up. The factor 1/2 is justified by the sum being necessarily an even number due to the double occurrence of the same pair (i,j) (in the scientific literature, this factor is either absent or replaced by ${\left(m-1\right)}^{-1}$). Consequently, the minimum deviation distance is 1, occurring in the case of a single swapping in a pair of contiguous positions, while the maximum one can be shown [11] to be $m^{2}/4$ if *m* is even and $(m^{2}-1)/4$ if *m* is odd. For m=10 as here considered, the maximal deviation distance is therefore 25. It is worth noting that this metric is preferable due to the presence of the weight |i−j|, which, whenever the corresponding $\delta_{u_{i},v_{j}}$ is one, is “naturally” proportional to the noise amplitude.

Let S(σ) be the set of words observed for a noise amplitude σ. The set S(0) is then the deterministic set of words, namely those observed for zero noise, which are henceforth considered the ancestors. We now scan the difference set D(σ)=S(σ)\S(0), looking for the words that are at deviation distance d=1 from possible ancestors. If a word W∈D(σ) has two or more ancestors, the “kinship” priority among these last is given to the most populated one (where the original population matters, i.e., prior to the absorption step that follows here). This ancestor “absorbs” the population of *W*, which is eventually removed from the process. Repeating the procedure by progressively increasing the deviation distance *d* up to its maximum value leads to a complete absorption of the population of the difference set D(σ) back into the set S(0).

**Figure 5:**
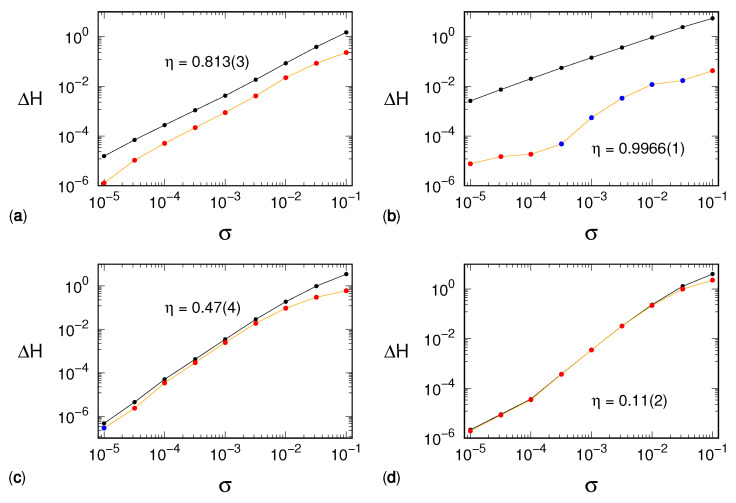
Results of the procedure of supervised distribution reconstruction: (**a**) Hénon map, (**b**) tent map, (**c**) generalized 3D Hénon map, (**d**) Mackey–Glass model. For each dynamical system, the black line corresponds to the plot ΔH(m,σ) that, as a function of Σ, is also reported in Figure 2. The orange line refers instead to the difference $\Delta H^{\prime }\left(m,\sigma \right)$ between $H^{\prime }\left(m,\sigma \right)$ and H(m,0), namely the entropy evaluated on the reconstructed histogram and the noiseless entropy, respectively. The related dots are colored in red (blue) if they represent a positive (negative) value of $\Delta H^{\prime }\left(m,\sigma \right)$. The value of the reconstruction efficiency η, evaluated via Equation (4) is also reported. The number in parentheses corresponds to the related uncertainty on the least significant digit.

While the procedure described above putatively puts back the amount of mass flown away because of the noise, it does not counter the democratization process of the original distribution. The entropy reduction is then expected to be partial and more effective, the larger the difference set D(σ). For this last reason, we expect the reconstruction to work better with the Hénon and tent maps than with the generalized 3D Hénon map and the Mackey–Glass model. The numerical results shown in Figure 5 confirm this prediction.

The figure shows the entropy increase $\Delta H^{\prime }\left(m,\sigma \right)$ after having coalesced the distribution on the set S(0) (i.e., subtracting H(m,0) from $H^{\prime }\left(m,\sigma \right)$). The efficiency of this strategy can be quantified by the parameter

(4) $$\eta =\frac{1}{\#\{\sigma \}}\sum_{\sigma }\frac{\Delta H\left(m,\sigma \right)-\Delta H^{\prime }\left(m,\sigma \right)}{\Delta H(m,\sigma )}\;,$$

where #{σ} is the number of noise amplitudes considered and such that both ΔH(m,σ) and $\Delta H^{\prime }\left(m,\sigma \right)$ are positive. The uncertainty on η is assessed as the standard deviation of the ratios. It is easily seen that if the method is perfectly efficient, i.e., if it cancels the noise-induced entropy increase, then η=1. On the contrary, if it is completely inefficient, it holds η=0. From the data reported in Figure 3, we see that the efficiency strongly declines when the dynamical complexity increases, being excellent for the tent map (η≲1) and, on the other hand, very modest for the Mackey–Glass model (η≈0.1). We further comment on this performance decline in the following section.

## 5. Discussion and Open Problems

In this paper, we have analyzed in a quantitative way the PE increase ΔH induced by a weak observational noise. We find that $\Delta H∼\Sigma^{k}$, where $\Sigma =\sigma m^{\alpha }$ can be interpreted as the effective amplitude of the noise (σ is the noise amplitude, and *m* is the length of the symbol sequence).

We have no theoretical arguments to justify the numerically assessed values of the α and *k* exponents, which turn out to be model-dependent. We are only able to qualitatively argue that they are related to the structure of the boundary of the set of deterministic (noiseless) sequences: the “longer” the boundary, the higher the set of new, spurious sequences generated by transposition errors induced by observational noise. In fact, we are only able to conjecture that α>1, so that a larger *m* value corresponds to a smaller distance between consecutive values of the sampled variable. In three out of the four models, α≈2.5, while α≈2 in the last one.

In order to shed some light on the growth of ΔH, we have studied the distribution of symbolic sequences according to their occurrence probability. The analysis allowed identifying a large number of poorly visited sequences, which are made accessible by noise. In the tent map and, to some extent, in the Hénon map, the diffusion of probability toward new, spurious sequences is responsible for a large fraction of ΔH. This is much less the case in the two other models. As discussed in the previous section, the elimination of the spurious sequences—at least via the algorithm proposed above—has proven to be inefficient in reconstructing the noiseless entropy whenever the dynamical complexity increases. A major reason appears to be the fact that “internal” diffusion among deterministic sequences is much stronger than the “external” diffusion toward new, spurious ones.

Quantifying the internal diffusion process is not easy because, given any two words $W_{a}$ and $W_{b}$ belonging to the deterministic set S(0), one should distinguish between noise-induced moves from $W_{a}$ to $W_{b}$ and vice versa. Since the entropy variation is due to population changes induced by the unbalance between incoming and outgoing fluxes, we have, therefore, opted for an indicator that can be easily estimated. To this purpose, we split all the words belonging to S(0) into two groups: the group of the words that increase their mass under the action of noise, and the group of those that lose mass. By denoting with $\Delta N_{G}$ the mass gain of the first group, and with $\Delta N_{L}$ the (positive) mass loss of the second one, it is clear that

(5) $$\Delta N_{L}=\Delta N_{G}+\Delta N_{\mathrm{external}}\;,$$

where the last term $\Delta N_{\mathrm{external}}$ stands for the mass migration toward the new sequences and is countered for by the method described in the previous section.

A possible proxy measure to assess the internal diffusion would then be $\Delta N_{G}$, whose entity can only stem from mass redistribution within S(0). However, it is more convenient to rely again on a relative measure. Hence, we propose to normalize $\Delta N_{G}$ by means of $\Delta N_{L}$ and to use the rescaled parameter

$$\rho_{\mathrm{in}}\equiv \frac{\Delta N_{G}}{\Delta N_{L}}$$

to quantify the relevance of the internal diffusion with respect to the global loss experienced by the first group. Due to Equation (5), it immediately follows that the complementary, “escape” parameter $\rho_{\mathrm{out}}$, defined as

(6) $$\rho_{\mathrm{out}}\equiv 1-\rho_{\mathrm{in}}=\frac{\Delta N_{\mathrm{external}}}{\Delta N_{L}}\;,$$

quantifies the relevance of the mass losses toward the set of the new, spurious sequences with respect to the global loss experienced by the first group. Values of $\rho_{\mathrm{in}}$ ($\rho_{\mathrm{out}}$) close to one (zero) hint at a dominating democratization progress that occurs within the deterministic set S(0). On the contrary, as $\rho_{\mathrm{in}}$ ($\rho_{\mathrm{out}}$) tends to zero (one), the escape process of the population out of the deterministic set S(0) progressively dominates.

Altogether, the numerical results discussed above suggest that the structure of the ensemble of noiseless sequences in the abstract space of ordinal patterns plays an important role. If the ensemble is fragmented and characterized by a long boundary, the effect of noise is mostly quantified by the emergence of new sequences. This is, for example, the case of the tent map. If the ensemble is compact and characterized by a short boundary, the effect of noise mostly leads to a redistribution of probabilities. This is what happens in the Mackey–Glass model.

**Figure 6:**
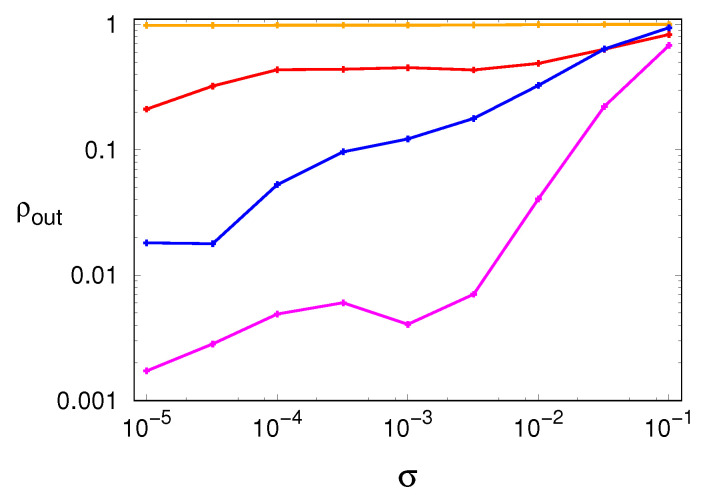
Escape parameter ρ, defined in Equation (6), as a function of the noise amplitude σ: (red) Hénon map, (orange) tent map, (blue) generalized 3D Hénon map, (magenta) Mackey–Glass model.

Figure 6 shows the plots of the escape parameter for all dynamical systems, confirming this last expectation as well as the interpretation provided above for the different kind of noise robustness shown by each dynamical system.

In general, we found that noise-induced diffusion among existing sequences represents a serious obstacle to the reconstruction of the noiseless PE. This is especially true for high-dimensional systems, where a large number of sequences are unavoidably visited, even in the absence of noise. Operating with a larger window length *m* would mitigate the problem (here, most of our simulations, refer to m=10), but at the cost of having to deal with a much longer time series so as to ensure enough statistics. A better strategy might be to restrict the analysis to the most populated sequences, which are definitely more robust to noise.

## Data Availability

Data sharing not applicable.
